# Anticorrosion Properties of a Novel Hybrid Sol–Gel Coating on Aluminum 3003 Alloy

**DOI:** 10.3390/polym14091798

**Published:** 2022-04-28

**Authors:** Rami K. Suleiman, Akeem Y. Adesina, Arumugam Madhan Kumar, Mohammad Mizanur Rahman, Fadi A. Al-Badour, Bassam El Ali

**Affiliations:** 1 Interdisciplinary Research Center for Advanced Materials, King Fahd University of Petroleum & Minerals (KFUPM), Dhahran 31261, Saudi Arabia; adesina@kfupm.edu.sa (A.Y.A.); madhankumar@kfupm.edu.sa (A.M.K.); mrahman@kfupm.edu.sa (M.M.R.); fbadour@kfupm.edu.sa (F.A.A.-B.); 2 Mechanical Engineering Department, King Fahd University of Petroleum and Minerals (KFUPM), Dhahran 31261, Saudi Arabia; 3 Chemistry Department, King Fahd University of Petroleum and Minerals (KFUPM), Dhahran 31261, Saudi Arabia; belali@kfupm.edu.sa

**Keywords:** aluminum, coatings, corrosion, roughness, sol–gel, waste

## Abstract

In this study, a novel hybrid sol–gel coating on AA3003 substrate was developed and the effects of various waste material additives on the reinforcement of the sol–gel coating and the anticorrosion properties in the saline medium were investigated. Egg shell, crumb rubber, activated carbon obtained for pyrolysis of waste rubber tire, waste rubber tire, cement kiln dust, and ST100 additives were tested as reinforcement materials. The AFM characterization results of the coating formulations on AA3003 alloy revealed enhanced roughness values for the modified coatings as compared to the base coating. Similarly, no significant changes were detected in the Fourier transform infrared spectroscopy (FTIR) absorption peaks of the hybrid polymeric material upon loading it with the waste additives, while slight changes in the hydrophobic properties of the final modified coatings were observed as a result of the modification process. Electrochemical impedance spectroscopy (EIS) results revealed that the hybrid sol–gel coating had a promising potential for the protection of the AA3003 substrate against corrosion in the saline medium. However, the loaded additives negatively affected the corrosion resistance properties of the parent hybrid sol–gel coating. For instance, the egg shell additive had the least negative effect on the barrier properties, whereas the cured coating layer of the sample loaded with cement and clay additives showed some disintegration, inhomogeneity, and low barrier properties on the metal surface.

## 1. Introduction

The degradation of metallic infrastructure caused by corrosion has long been a challenge to various human life sectors. Aluminum–manganese alloy AA3003 is commonly used for roofing, gutters, and sidings, and exhibits desired properties such as moderate strength, good workability, and good corrosion resistance [1]. Although the oxide layer that forms on the surface of aluminum (Al) alloys can have a protective effect against corrosion, it can still be breached in aggressive environments, such as NaCl-containing environments, leading to the formation of pits on the Al surface [2,3]. Chemical conversion coatings have been widely used to protect Al surfaces against corrosion, as well as to enhance the adhesion properties of the primer coating to the surface. However, due to the health concerns related to the toxicity of such conversion coatings, especially chromium(VI)-based coatings, several replacements have been proposed in the literature [4].

In addition to the use of Cr(VI)-free anodization, nanocomposites, and metal-rich primers, hybrid sol–gel coatings have been studied extensively in the literature as one of the promising alternative mitigation approaches for the corrosion of Al alloys in aggressive environments [5,6,7,8,9]. These hybrid polymeric materials are prepared via the hydrolysis of metal alkoxides and organoalkoxysilane precursors and provide the combined advantageous properties of both organic (hydrophobicity, elasticity, and others) and inorganic (stiffness, thermal stability, inertness, etc.) coatings [10,11]. In comparison to other Al alloys, the electrochemical, mechanical, and morphological performances of hybrid sol–gel coatings on AA3003 have been less studied in the literature.

For example, Gobra studied the effects of TiO_2_/SiO_2_ nanoparticle additives on the mechanical properties of a hybrid silica sol–gel epoxy coating applied to an AA3003 substrate. Significant improvements in the adhesion and hydrophobicity of the hybrid coating were observed as a result of the SiO_2_ addition [12]. In another study by Niknahad and Mannari, the sol–gel methodology was used to fabricate hybrid nanocomposite films starting from a bis-ureasil silane precursor and colloidal nanoparticles. The synthesized coating films proved to be a very effective and sustainable pretreatment for AA3003 H14 alloy [13]. Subasri et al. also implemented an in situ anodization approach along with sol–gel deposition methodologies for the preparation of dense, thick, and stable silica–zirconia coatings on AA3003 alloy. The composite aluminosilicate coating layer doped with Ce^3+^ additive was found to have good barrier properties against corrosion [14]. Whelan et al. also reported a great influence of the extent of sol–gel penetration and pore sealing on the anticorrosion properties of different anodized aluminum AA3003 layers in a neutral salt spray and 3.5% *w*/*v* solution of NaCl(aq) [15]. The above studies and many others on hybrid sol–gel coating systems applied to other metallic substrates have proven the need for methodologies to overcome the presence of microcracks and small pores or pinholes in the micro- and nanostructures of sol–gel coatings; these defects can facilitate the passage of aggressive corrosive ions through the coating–substrate interface, leading to corrosion of the subsurface [16,17,18]. These methodologies should involve either the alteration of the sol–gel structures and management of their properties by varying the precursor structure and sol–gel processing conditions [19,20,21], or the embedment of advantageous additives such as fillers, corrosion inhibitors, and biocides in the parent hybrid sol–gel coating systems [12,22,23,24]. In particular, additives derived from materials used in daily life, the residues of which are considered waste materials, are promising and economic components of various classes of coatings. For example, Adesina et al. reported enhanced mechanical and tribological properties of a composite epoxy coating embedded with 1–20 wt.% micronized waste tire rubber [25]. Nanocomposites containing different amounts of cloisite 20A (C20A) nanoclay additive were also successfully prepared and reported. A coating loaded with 3 wt.% C20A showed higher adhesion strength and corrosion protection compared to a neat coating and other percentages of C20A in the nanocomposites [26]. In another study, Najafisayar et al. investigated the effects of an egg shell bio-additive on the corrosion and wear properties of anodic films produced on anodized AA1050 aluminum alloy [27]. Scarce information is available in the literature on the interactions of such waste additives with hybrid sol–gel coatings systems; hence, this area of research is challenging and open for development.

The current work examines the anticorrosion performance of a novel hybrid coating on AA3003 panels, as well as the impacts of various waste material additives on the morphological, thermal, and barrier properties. The surface properties of the modified coatings are investigated via AFM analyses. The anticorrosion behaviors of the neat and additive-embedded hybrid coatings on Al substrates are evaluated after a 30-day immersion period in a 3.5 wt.% NaCl electrolytic solution.

## 2. Experimental Section

### 2.1. Materials

Aminopropyltriethoxysilane (APTES), methyltrimethoxysilane (MTMS), 1,2-epoxybutane (EB), and isopropyl alcohol (IPA) were obtained from Sigma-Aldrich, St. Louis, MO, USA. Vinyltrimethoxysilane (VTMS), 3-isocyanatopropyltriethoxysilane (ICPTES), and polydimethylsiloxane (PDMS), in silanol-terminated form and with a molecular weight range of 400–700, were purchased from Gelest Company (Morrisville, PA, USA). All chemicals utilized in this study were of analytical grade and used without further purification. The waste material additives were acquired from various local sources.

### 2.2. Synthesis of the Host Hybrid Coating

The neat hybrid polymeric sol–gel material (named after “S”) was prepared by mixing 10 mL of each of the precursors EB (8.29 g, 0.115 mol), APTES (9.46 g, 0.043 mol), MTMS (9.55 g, 0.070 mol), VTMS (9.68 g, 0.065 mol), and PDMS as a solution for 2 h. The hydrolysis and polycondensation reactions of the silanol groups of the silane mixture were achieved via the dropwise addition of 1 mL of a 2:1 mixture of isopropyl alcohol (IPA)–0.05 N HNO_3_ under continuous stirring. The resulting solution was allowed to react with 10 mL of the precursor ICPTES (9.50 g, 0.040 mol) for 10 min in a beaker at room temperature (RT). To this solution, 10 mL of VTMS (9.68 g, 0.065 mol), 10 mL of PDMS solution, and PI (0.1 g, 0.489 mmol) were then added to the reaction mixture. Finally, 2 mL of the IPDI precursor (2.098 g, 0.009 mol) and the resultant colorless solution were stirred at 350 rpm for 24 h on a magnetic stirrer. The homogeneous polymeric solution was aged for 1 day in the open air at RT prior to its functionalization with waste material additives.

**Figure 1:**
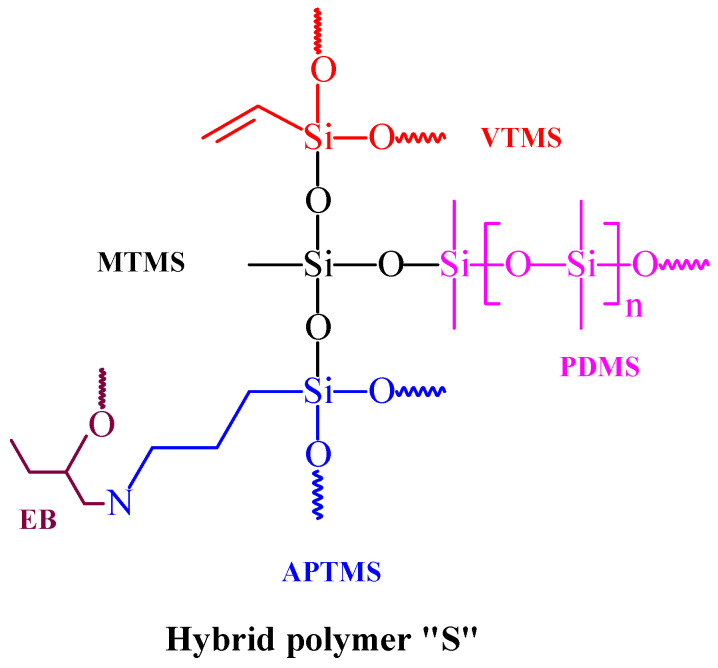
Proposed sol–gel networks in the hybrid polymer “S”.

The synthesis of the hybrid polymer “S” involved a cross-linking coupling reaction between the EB and APTES precursors, which was designed to enhance the interfacial interactions between the inorganic and organic components of the hybrid polymer. The formation of the sol–gel network was achieved via the catalytic hydrolysis of the APTES, MTMS, and VTMS silane precursors in the presence of an acid–alcohol catalyst, then further densification was also achieved by adding the PDMS agent to the reaction mixture. This reagent is proven to introduce more scratch resistance and hydrophobic properties to the coating matrices [28]. The addition of IPA to the acid–water solution helps in enhancing the miscibility of the silane precursors and water, enhancing the homogeneity of the polymer solution [29]. A proposed chemical structure for the hybrid sol–gel networks that might exist in the polymer “S” is depicted in Figure 1.

### 2.3. Formulation of the Waste-Additive-Embedded Coatings

The waste-additive-functionalized coatings were prepared by adding 0.5 g of various micronized waste materials (particles size: 50–100 μm; activated carbon (**S-AC**), cement kiln dust (**S-CE**), clay nanoparticles (**S-CL**), egg shells (**S-EG**), rubber powder (**S-RB**), and ST100 powder (**S-ST**)) individually to 10 mL of the parent coating (**S**), then the resulting solution was sonicated using an ultrasonic probe (Vibracell, Sonics, Newtown, CT, USA) for 5 min. The mixing time between the additive and the parent coating was carefully optimized in order to avoid any gelification of the coating solution prior to its application to the metal surface.

### 2.4. Pretreatment of Aluminum Alloy Substrates

The commercially available 3003-H14 aluminum Q-panels (part no. Al-36, Q-Lab Company, Westlake, OH, USA, 3 × 6 × 0.025-inch dimension) (chemical composition in wt.%: Mn (1.00–1.50), Fe (0.70), Si (0.60), Zn (0.10), others (0.05/0.15), and Al (remaining)) was used as a metallic substrate. The surface of the Al panels was washed with IPA and finally dried with a hot air blower prior to the application of the coating formulations.

### 2.5. Deposition of Functional Coatings

**Figure 2:**
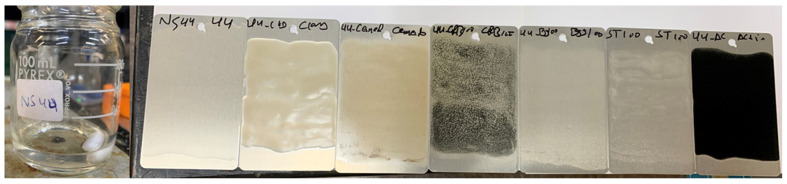
Physical appearances of the neat hybrid polymer (left) and the cured coating formulations on AA3003 substrate.

The S-coating matrices were deposited on the dried Al panels using a rod applicator (K101 control coater, Hertfordshire, UK), and the resultant uniform coatings on Al were cured in an oven at 100 °C for 24 h. Approximate uniform and controlled dry coatings of about 50 μm in thickness (measured using a PosiTector 200 coating thickness cage, Buford, GA, USA) were formed on the panel surface. The surfaces of the cured coating layers of all formulations on the Al surface were homogeneous, crack-free, integrated, and with no sign of coating delamination (Figure 2).

### 2.6. Characterization of Coatings

The chemical compositions of the S-coating formulations were analyzed using a Thermo Scientific Nicolet IS5 Fourier transform infrared spectrometer, where the Attenuated total reflection (ATR) was used in the observation range of 600–4000 cm^−1^. The surface topography of the AA3003-coated panels was examined using an AFM (MFP-3D Origin, Oxford Instruments, Abingdon, UK). The thermal degradation behavior of the cured coating layers on AA3003 substrates was investigated using the TGA 1 STAR system (Mettler Toledo, Columbus, OH, USA) under ambient atmospheric conditions at a heating rate of 10 °C/min up to 800 °C.

The anticorrosive performance assessment of the S-coating formulations on AA3003 substrates after 30 days of exposure to 3.5 wt.% NaCl electrolytic solution was performed using the electrochemical impedance spectroscopy (EIS) measurements (GAMRY Reference 3000 potentiostat and galvanostat, Warminster, PA, USA). The obtained EIS data of the coated samples were simulated using the Echem Analyst software (version 6.04). The EIS measurements were conducted at the open circuit potential (OCP), a frequency range of 100 kHz–10 mHz, and a perturbation voltage of 10 mV. These experiments were conducted in a typical three-electrode cell with the coated AA3003 sample used as a working electrode, a graphite rod as the counter electrode, and the saturated calomel electrode (SCE) as the reference electrode. The test area of the sample was 10 cm^2^.

## 3. Results and Discussion

### 3.1. Structural Analysis of the Functional Coatings

**Figure 3:**
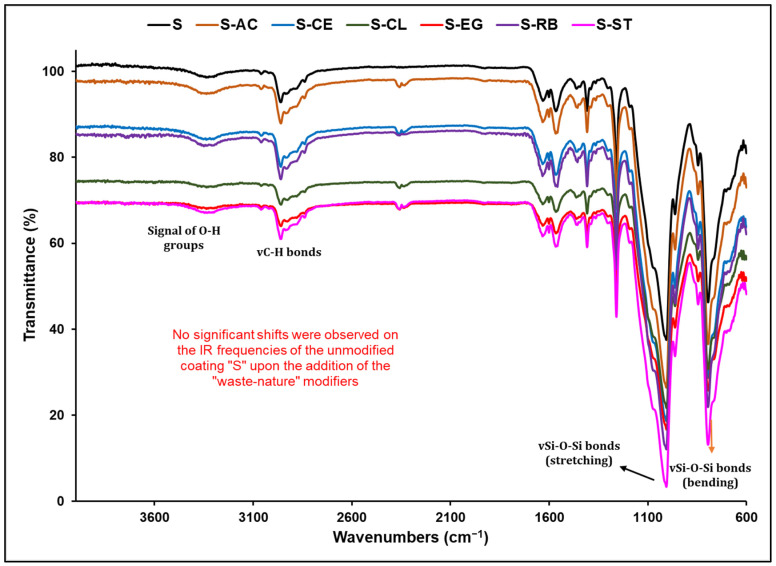
FTIR spectra of the unmodified and waste-material-modified hybrid sol–gel polymers.

The study of the structural characteristics of the hybrid polymer “S”, as well as any structural changes that might have resulted from loading it with the waste material additives, was performed via the Fourier transform infrared spectroscopy (FTIR) analysis. Figure 3 shows the FTIR spectra of the parent and waste-loaded coating formulations. The IR peaks in the range of 2900–3000 cm^−1^ in all spectra are assigned to the characteristic CH_3_ and CH_2_ moieties of the various silane precursors, while the broad band at around 3350 cm^−1^ is attributed to the stretching vibration of the O-H or N-H functionalities in the polymer network and the H-O-H bending vibration of water [30]. The broadening of this peak resulted possibly from the formation of a hydrogen bond between the two above-mentioned functionalities and water. The presence of two strong peaks at around 1006 and 1078 cm^−1^ confirmed the presence of two types of Si-O-Si networks, in which one is resulted from the hydrolysis and polycondensation of the silane precursors and the existing PDMS precursor [31]. The peak at 979 cm^−1^ is also associated with the Si-O bond vibrations. The above assignments prove the formation of the hybrid polymer “S”. A comparison of the spectra of the additive-loaded coatings with the spectrum of the base “S” coating revealed great similarity between the two groups of spectra, indicating a lack of any chemical interactions between the additives and the hybrid sol–gel polymeric network, indicating the composite structure of the final functional coatings.

### 3.2. Gravimetric Thermal Analysis of the Cured Functional Coatings

**Figure 4:**
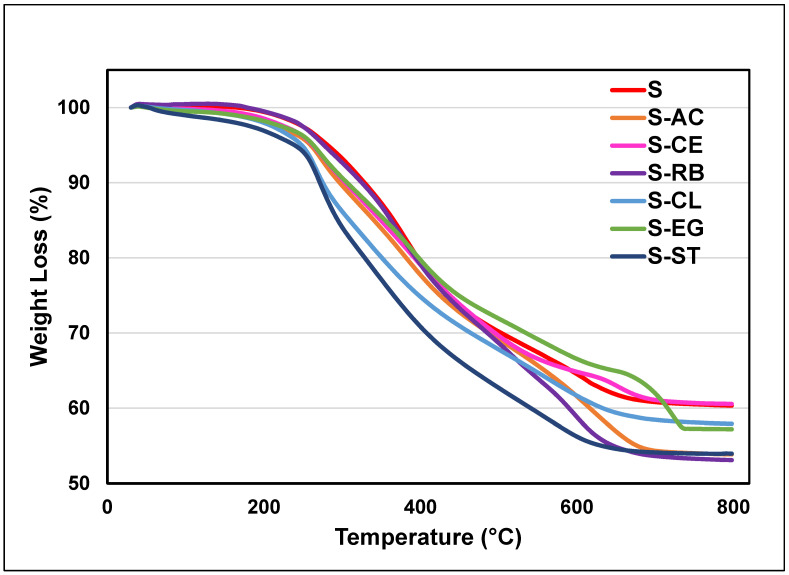
TGA plots of the S-coating matrices.

The thermogravimetric analysis (TGA) was carried out on the developed coatings in this study in order to investigate the impacts of adding the waste material additives on the thermal properties of the parent “S” coating (Figure 4). This technique is more valuable in terms of providing information on the volatile compositions and characteristic decomposition patterns of polymers [32]. The TGA thermograms depicted in Figure 4 indicate the occurrence of a slight degradation in the range of 150 to 220 °C resulted possibly from the evaporation of water and IPA components and the condensation of the Si-OH side groups of the hybrid polymer [23]. More significant changes in the TGA profiles of the coating formulation can be observed in the temperature range of 220–650 °C, which can be attributed to the degradation of the organic components of the coatings [33]. The degradation process was completed at a temperature of 800 °C, and one can see the relative thermal stability of the hybrid coatings at this high-temperature range. Moreover, careful analysis of the TGA thermograms of the modified and unmodified coating formulations on AA3003 (Figure 4) reveals minor effects of the type of loaded waste material additive on the degradation degree and pattern of the parent hybrid polymeric network.

### 3.3. Surface Morphology of the Coating Formulations on Aluminum

The hydrophobic and hydrophilic properties of the various coating matrices on the AA3003 surface were characterized by the aqueous contact angle (CA) measurements using the sessile drop method. As can be noticed from the CA data listed in Table 1, a hydrophobic nature can be assigned to S, S-AC, S-CL, and S-RB samples, while the surfaces of the remaining samples exhibit a relatively hydrophilic property. In general, enhancing the hydrophobic properties of a parent coating formulation on metal surfaces via the addition of coating additives is usually beneficial to the corrosion resistance properties of the coating system, unless this modification step also leads to deterioration of the integrity, homogeneity, or adherence properties of the modified coating [34]. The slight change in the water CA values of the waste-additive-modified coatings on AA3003 panels can be attributed to the degree of physical interaction between the additive and the hybrid sol–gel polymeric network, and also to the morphologies of the cured functionalized coatings on the aluminium surface [17]. Sample S-CE has the lowest CA value compared to other modified coatings, which can be attributed to the presence of strong interaction forces between water and the cement additive particles.

**Figure 5:**
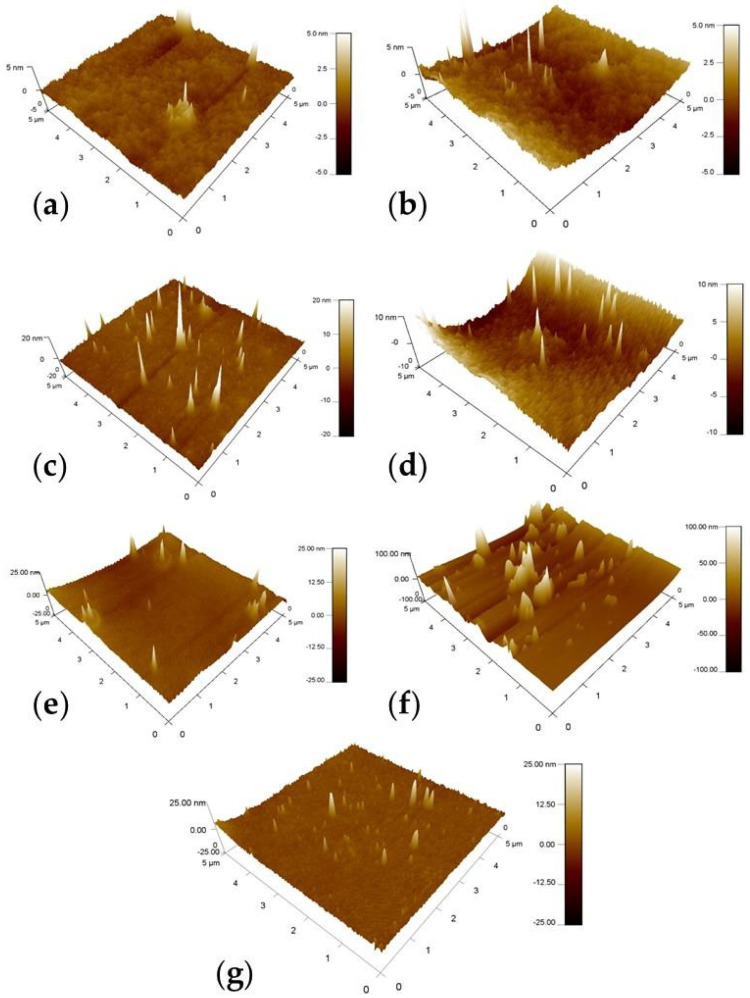
AFM micrographs of (**a**) S-, (**b**) S-AC-, (**c**) S-CE-, (**d**) S-CL-, (**e**) S-EG-, (**f**) S−ST-, and (**g**) S-RB-coated samples.

The surface roughness results for the fabricated coatings on the AA3003 surface were obtained from the AFM test. Figure 5 depicts the AFM tridimensional (3D) images obtained from the free surface of the sample and registered at the scale of 5 × 5 µm^2^, while Table 2 lists the roughness parameters for the composite hybrid coatings. The R_a_ parameter represents the arithmetic average height of the surface, R_ms_ represents the root mean square roughness, and R_pv_ represents the difference between the lowest and highest points of the scanned area (peak-to-valley difference) [35]. A comparison between the surface roughness values of the parent “S” Al-coated sample and the additive-loaded samples in Figure 5 reveals that the loading step results in increases in the height variation parameters and in the surface roughness, leading to 0.41 to 17.64 nm increases in R_a_ values. Nevertheless, the relatively small R_a_ values for all the coating matrices indicate excellent co-solubility behavior for all loaded additives with the bulk of the parent hybrid sol–gel polymer. Overall, sample S-ST exhibited the highest surface roughness behavior, which resulted from the film irregularities that can be seen in Figure 5f, whereas sample S-AC (Figure 5b) showed the lowest R_a_ value, indicating the high uniformity of this coating layer on the AA3003 surface. Although sample S-CE showed a relatively low R_ms_ value, its R_pv_ value was slightly high (compared with other samples S-AC and S-CL), indicating a lesser degree of coating film smoothness in this sample (Figure 5c).

### 3.4. Corrosion Behavior of the Hybrid Coating Formulations on Aluminum Substrate

**Figure 6:**
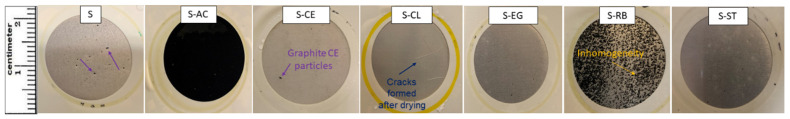
Digital photo images of the Al-coated samples after exposure to the 3.5 wt.% NaCl for 30 days.

The anti-corrosion performances of the developed coating formulations on AA3003 samples continuously exposed to 3.5 wt.% NaCl electrolytic solution were evaluated using visual and electrochemical impedance spectroscopy (EIS) techniques. Figure 6 depicts the digital photo images of the Al-coated samples after exposure to the 3.5 wt.% NaCl for 30 days. Overall, the parent and additive-loaded coating formulations exhibited excellent corrosion protection performance for the aluminum surfaces during this relatively long immersion period. In this aspect, no major defects, macropores, or delamination phenomena could be seen on the surfaces of these samples. However, sample S-CL showed some cracks in the coating film after removing the chloride solution, indicating the presence of competing low-barrier properties for this sample.

**Figure 7:**
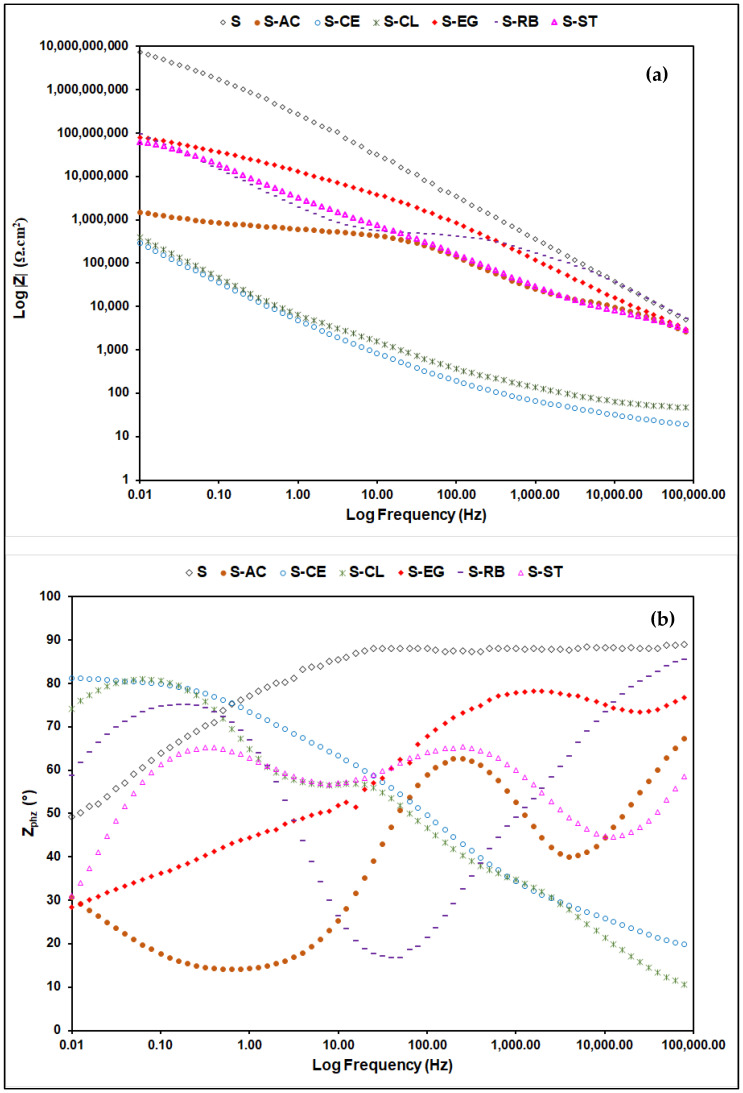
(**a**) EIS and (**b**) Bode phase plots of all coated samples on AA3003 substrate after 30 days of exposure to the 3.5 wt.% NaCl solution.

**Figure 8:**
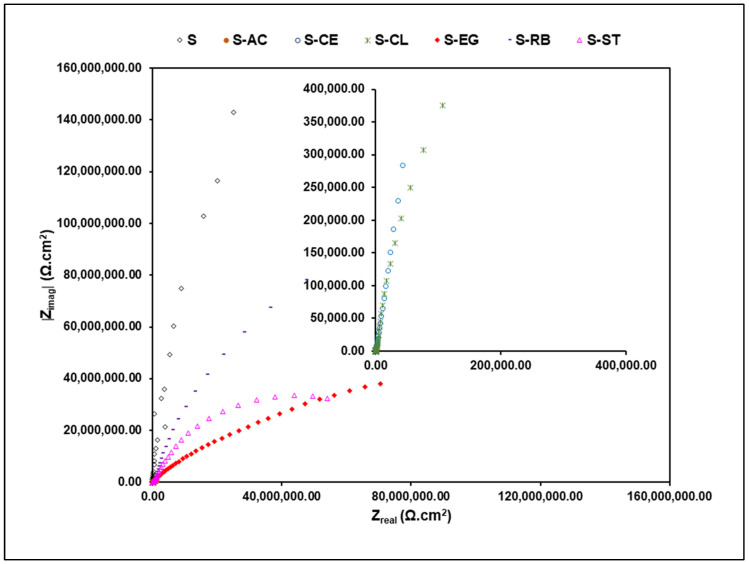
EIS Nyquist spectra of all coated samples on AA3003 substrate after 30 days of exposure to the 3.5 wt.% NaCl solution.

The aforementioned visual observation results were complemented by the conductance of EIS analysis on the coated samples. For the EIS experiments, the aluminum panels coated with the parent “S” and the additive-modified coatings were exposed continuously to 3.5 wt.% NaCl, and the EIS data were collected after 30 days of exposure and plotted as Bode modulus and phase angle values (Figure 7) and Nyquist (Figure 8) plots. The high-frequency regions of the Nyquist curves were enlarged in order to achieve a clearer visualization of the EIS behavior of the tested samples. EIS is one of the most informative techniques for studying the electrochemical behavior of coating systems [11,36,37]. As can be seen from Figure 7a, samples S, S-AC, S-EG, S-RB, and S-ST exhibited impedance values higher than 10^6^ Ω·cm^2^, indicating coatings of high integrity, less porosity, and excellent barrier properties on the aluminum surface [17], whereas samples S-CE and S-CL showed relatively low impedance values resulting possibly from the inhomogeneity of the coating films of these sample on the aluminum surface. Moreover, impedance values for the additive-modified formulations were found to be less than those of the “S” sample. This refers to the disadvantageous role of the embedded waste materials in these samples in deteriorating the barrier properties of the bulk “S” sample. The Bode phase (Figure 7b) and Nyquist (Figure 8) plots of the EIS data for all Al-coated samples clearly showed the presence of three-time constants. The one in the high-frequency range is associated with the capacitance and pore resistance of the hybrid film, the one in the middle-frequency range is associated with the properties of the oxide layer on the surface, and the last one at low frequencies is typically attributed to the electrochemical corrosion processes on the metal surface [38]. From the Nyquist plot depicted in Figure 8, samples S, S-AC, S-EG, S-RB, and S-ST have wider semicircles compared with other samples, indicating better barrier properties for these samples against the passage of corrosive ions on the metal surface [10]. The Nyquist plot of each individual coated sample is available in the Supporting Information.

**Figure 9:**
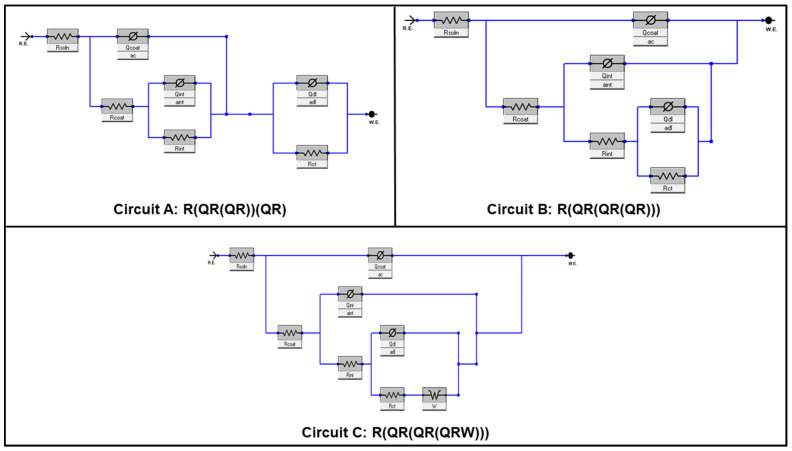
Electrochemical equivalent circuits used in fitting the EIS data of the coating matrices on AA3003 substrate.

The three equivalent circuit (EC) models presented in Figure 9 were tested when fitting the EIS spectra. These circuits were selected by taking into consideration the existence of a physical and high-quality fitting output for the tested circuits. The circuits **A** [R_soln_(Q_coat_R_coat_(Q_int_R_int_))(Q_dl_R_ct_)] [11], **B** [R_soln_(Q_coat_R_coat_(Q_int_R_int_(Q_dl_R_ct_)))] [17], and **C** [R_soln_(Q_coat_R_coat_(Q_int_R_int_(Q_dl_R_ct_W)))] [38] were typically implemented in describing the EIS data of hybrid sol–gel systems and organic coatings on metallic substrates, and their suitability in describing the EIS data of the current work was investigated and compared.

In these circuits, the first element R_soln_ corresponds to the solution resistance, which is important to improve the fitting goodness. The [Q_coat_R_coat_] element is attributed to the sol–gel coating capacitance and resistance, while the [Q_dl_R_ct_] component accounts for the corrosion process that might develop during immersion in the corrosive solution and is characterized by the double-layer capacitance and charge transfer resistance at the coating–metal interface. The third component [Q_int_R_int_] characterizes the aluminum oxide film porosity and dielectric properties formed at the metal–coating interface. The use of the constant phase element (CPE), represented by Ø in circuits shown in Figure 9, instead of an ideal capacitor was found to be advantageous for the fitting quality. This element is widely employed in EIS fitting to account for the inhomogeneity of double layers and the dispersive nature of time constants [39]. The degree of deviation of the capacitance from the ideal capacitor is represented by the CPE exponent “*n*”, with values ranging from *n* = 1 for an ideal capacitor to *n* = 0.5 for a Warburg element (*W*) and *n* = 0 for a resistor. The quality of the fit (goodness of fit) between the calculated and experimental EIS data can be assessed by observing the obtained chi-square (χ^2^) value from the fitting, where values of 10^−3^ for χ^2^ and lower indicate a relatively high-quality fit [40]. Moreover, the trend between the calculated Q_dl_ and R_ct_ values should agree with the corrosion protection ranking obtained experimentally for the coated samples. Considering the above factors, the values of Qdl, Rct, and χ^2^ of S-coated samples fitted using equivalent circuits A–C are listed in Table 3.

The fitted parameter values depicted in Table 3 indicate the suitability of circuit B for samples S, S-EG, S-RB, and S-ST, while circuit A better fit the EIS data of samples S-CE and S-CL. Circuit C was adopted only in the fitting of the EIS data of sample S-AC, as it was the only sample with a capacitive arc of 45° in its Nyquist spectrum (Figure S2, Supporting Information). In addition to the goodness of fit criteria, the obtained fitted values for the Qdl and Rct elements were in full agreement with the corrosion resistance ranking of all coated samples. These parameters are very important in evaluating the corrosion protection performance of hybrid sol–gel coatings exposed to corrosive media. The lack of ability of circuits B and C to properly fit the EIS data of sample S-CE vividly supports the importance of the quality of the fitting issue, whereas the physical aspect of the employed circuit received our full consideration during the fitting of EIS data of the S-AC sample, where even low chi-square values were produced.

Table 4 lists the fitted parameter values of the EIS data for all coated samples using proper equivalent circuits. The data clearly indicated that the parent hybrid sol–gel coating has a highly water-repellent and insulating nature, which provided a strong barrier to the corrosive ions that prevented the electrolytes from reaching the aluminum substrate. Therefore, a significant part of the impedance results from the solution resistance, the coating resistance, and the coating capacitance [41]. Moreover, the electrostatic negative charge of the OH^−^ anions that might form at the electrolyte–coating interface can exhibit a repulsive electrostatic force that can retard the diffusion of Cl^−^ ions from the bulk to the interface, in turn inhibiting the formation of the [AlCl_6_]^3−^ complex caused by pitting of the Al surface [42]. Samples S-CE and S-RB exhibited high R_coat_ values, revealing the nonporous nature of the two coating formulations.

Considering both the visual and EIS testing results, the corrosion protection capabilities of the “S” hybrid coating can be expected for AA3003 substrate in real saline environments.

## 4. Conclusions

A hybrid sol–gel composite coating for protecting AA3003 substrates was developed and its corrosion resistance was evaluated. Reinforcements with 5 wt.% of different waste material additives were successfully applied. The EIS results for the exposed AA3003 panels coated with 3.5 wt.% NaCl solution revealed that the loaded additives affected the anticorrosion performance of the base hybrid coatings to different extents, related primarily to the degree of homogeneity between the additive and coating matrix, as proven by the AFM analyses. The egg shell additive was found to be well-dispersed in the parent coating matrix, which reduced the passage of the corrosive chloride and oxygen ions to the aluminum surface. The hybrid sol–gel coating formulation prepared in this study can be considered a potential replacement for the conventional chromate-based conversion coatings.

## Figures and Tables

**Table 1:** Water contact angle (CA) values (in °) of the cured coatings on AA3003 substrate.

Sample	CA (°, Mean ± SD *)
**S**	94 ± 1
**S-AC**	91 ± 3
**S-CE**	81± 1
**S-CL**	95 ± 4
**S-EG**	85 ± 2
**S-RB**	93 ± 1
**S-ST**	87 ± 2

* SD: Standard deviation.

**Table 2:** Roughness parameters of the composite coatings.

	R_a_ (nm)	R_ms_ (nm)	R_pv_ (nm)
**S**	0.40	0.87	37.53
**S-AC**	0.81	1.16	29.34
**S-CE**	1.03	2.01	62.04
**S-CL**	3.32	5.34	59.64
**S-EG**	1.61	2.47	42.82
**S-RB**	0.93	1.43	30.14
**S-ST**	18.05	21.44	96.34

**Table 3:** Fitted Qdl (nF cm^−2^ s^−(1−αc)^), RCT (MΩ), and χ^2^ (goodness of fit, ×10^3^) parameter values of S-coated samples of EC presented in Figure 9.

Sample	Circuit A			Circuit B			Circuit C		
	Qdl	RCT	χ^2^	Qdl	RCT	χ^2^	Qdl	RCT	χ^2^
**S**	64.53	2.24	**1.20**	**3.77 × 10^−3^**	**625.20 × 10^3^**	**1.21**	NP	NP	NP
**S-AC**	NP	NP	NP	NP	NP	NP	**333.30**	**0.11**	**0.21**
**S-CE**	**45.09 × 10^3^**	**23.78**	**2.23**	NF	NF	NF	NF	NF	NF
**S-CL**	**0.034**	**1.91**	**0.12**	0.014	6.98	2.77	NP	NP	NP
**S-EG**	9.01	1.33	1.80	**27.42**	**422.00**	**1.86**	NP	NP	NP
**S-RB**	105.50	249.60	6.78	**105.60**	**250.10**	**6.78**	NP	NP	NP
**S-ST**	75.84	87.96	1.50	**22.13**	**105.20**	**1.34**	NP	NP	NP

NF: No possible fitting. NP: No physical meaning for the circuit.

**Table 4:** EIS circuit fitted parameters for the S-coating matrices on AA3003 substrate after 30 days of exposure to 3.5 wt.% NaCl solution.

Parameter	Sample						
	S	S-AC	S-CE	S-CL	S-EG	S-RB	S-ST
**Rsoln (Ω)**	10.00	26.40	15.00	40.21	22.00	50.00	18.83
**Rcoat (kΩ)**	11.50 × 10^3^	14.45	12.03	0.499	4.25 × 10^3^	453.90	19.18
**Qcoat** **(nF cm^−2^ s^−(1−αc)^)**	507.20 × 10^−3^	3.46	0.39	0.049	4.02	2.64	44.93
**ac**	0.98	0.88	0.45	0.58	0.87	0.85	0.68
**Rint (MΩ)**	2.78	0.49	0.155	0.0030	0.0026	250.10	2.26
**Qint (nF cm^−2^ s^−(1−αc)^)**	3.83 × 10^−3^	23.38	3.42 × 10^3^	7.53 × 10^3^	5.91	105.60	7.22
**aint**	0.44	0.86	0.51	0.89	0.83	0.88	0.91
**Rct (MΩ)**	625.20 × 10^3^	0.11	23.78	1.91	422.00	250.10	105.2
**Qdl (nF cm^−2^ s^−(1−αc)^)**	3.77 × 10^−3^	333.30	45.09 × 10^3^	0.034	27.42	105.60	22.13
**adl**	0.83	1.00	0.92	0.95	0.36	0.19	0.90
**W (μF cm^−2^ s^0.5^)**	-	3.74	-	-	-	-	
**χ^2^ (× 10^3^)**	1.21	0.21	2.23	0.12	1.86	6.78	1.34
**Circuit**	B	C	A	A	B	B	B

## Data Availability

The data presented in this study are available on request from the corresponding author.

## Supplementary Materials

The following supporting information can be downloaded at: https://www.mdpi.com/article/10.3390/polym14091798/s1, Figure S1. EIS Nyquist spectrum of S-coated samples on AA3003 substrate after 30 days of exposure to the 3.5 wt.% NaCl solution. Figure S2. EIS Nyquist spectrum of S-AC-coated samples on AA3003 substrate after 30 days of exposure to the 3.5 wt.% NaCl solution. Figure S3. EIS Nyquist spectrum of S-CE-coated samples on AA3003 substrate after 30 days of exposure to the 3.5 wt.% NaCl solution. Figure S4. EIS Nyquist spectrum of S-CL-coated samples on AA3003 substrate after 30 days of exposure to the 3.5 wt.% NaCl solution. Figure S5. EIS Nyquist spectrum of S-EG-coated samples on AA3003 substrate after 30 days of exposure to the 3.5 wt.% NaCl solution. Figure S6. EIS Nyquist spectrum of S-RB-coated samples on AA3003 substrate after 30 days of exposure to the 3.5 wt.% NaCl solution. Figure S7. EIS Nyquist spectrum of S-ST-coated samples on AA3003 substrate after 30 days of exposure to the 3.5 wt.% NaCl solution.
