# Supplementary material for: Anticorrosion Properties of a Novel Hybrid Sol–Gel Coating on Aluminum 3003 Alloy
Source: Polymers (Basel). 2022 Apr 28;14(9):1798. doi: 10.3390/polym14091798 (PMC9103624; doi:10.3390/polym14091798)
Supplement: Supplementary file 1 [file polymers-14-01798-s001.zip › polymers-1701610-supplementary.pdf]

polymers

Supplementary

Anticorrosion Properties of a Novel Hybrid Sol-Gel Coating on Aluminum 3003 Alloy

Rami K. Suleiman [1,*], Akeem Y. Adesina [1], Arumugam Madhan Kumar [1], Mohammad Mizanur Rahman [1], Fadi A. Al-Badour [1,2] and Bassam El Ali [3]

1. Interdisciplinary Research Center for Advanced Materials, King Fahd University of Petroleum & Minerals (KFUPM), Dhahran 31261, Saudi Arabia; adesina@kfupm.edu.sa (A.Y.A.); madhankumar@kfupm.edu.sa (A.M.K.); mrahman@kfupm.edu.sa (M.M.R.); fbadour@kfupm.edu.sa (F.A.A.-B.)
2. Mechanical Engineering Department, King Fahd University of Petroleum and Minerals (KFUPM), Dhahran 31261, Saudi Arabia
3. Chemistry Department, King Fahd University of Petroleum and Minerals (KFUPM), Dhahran 31261, Saudi Arabia; belali@kfupm.edu.sa

* Correspondence: ramismob@kfupm.edu.sa; Tel.: +966-13-860-7580; Fax: +966-13-860-8317

Figures

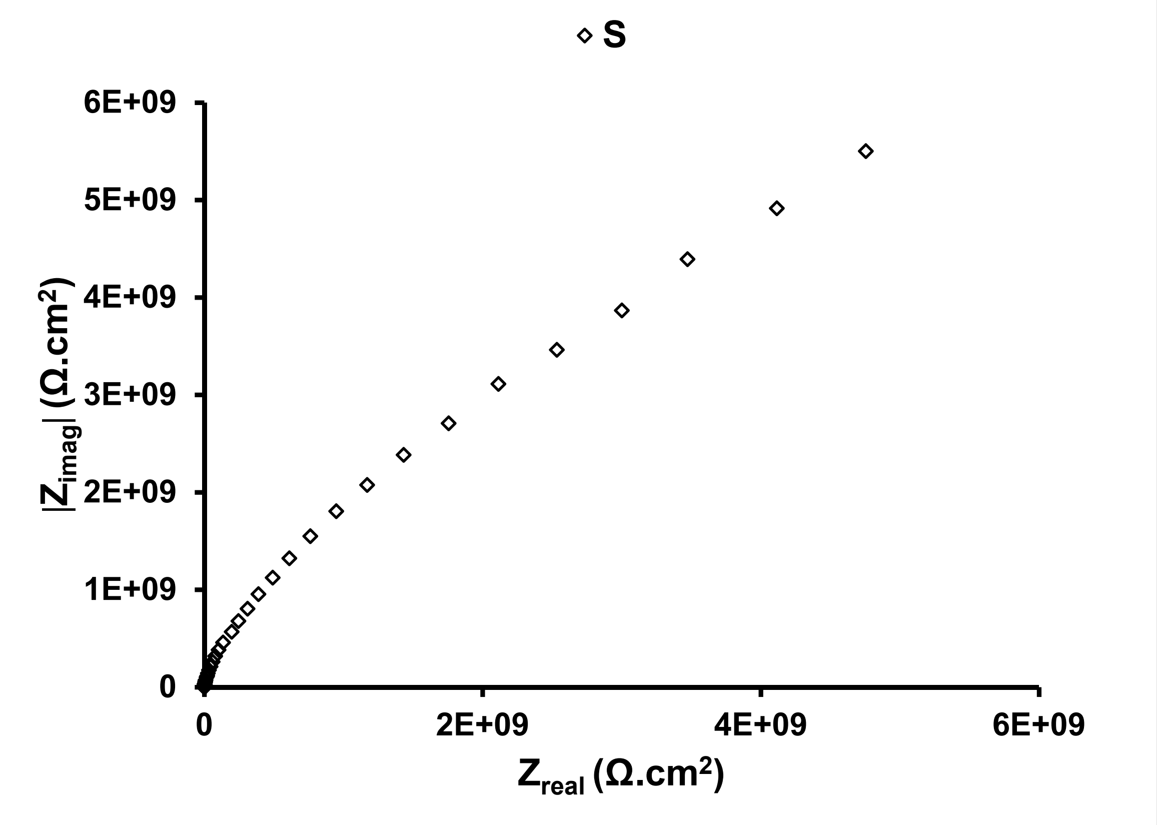

Figure S1. EIS Nyquist spectrum of **S**-coated samples on AA3003 substrate after 30 days of exposure to the 3.5 wt.% NaCl solution.

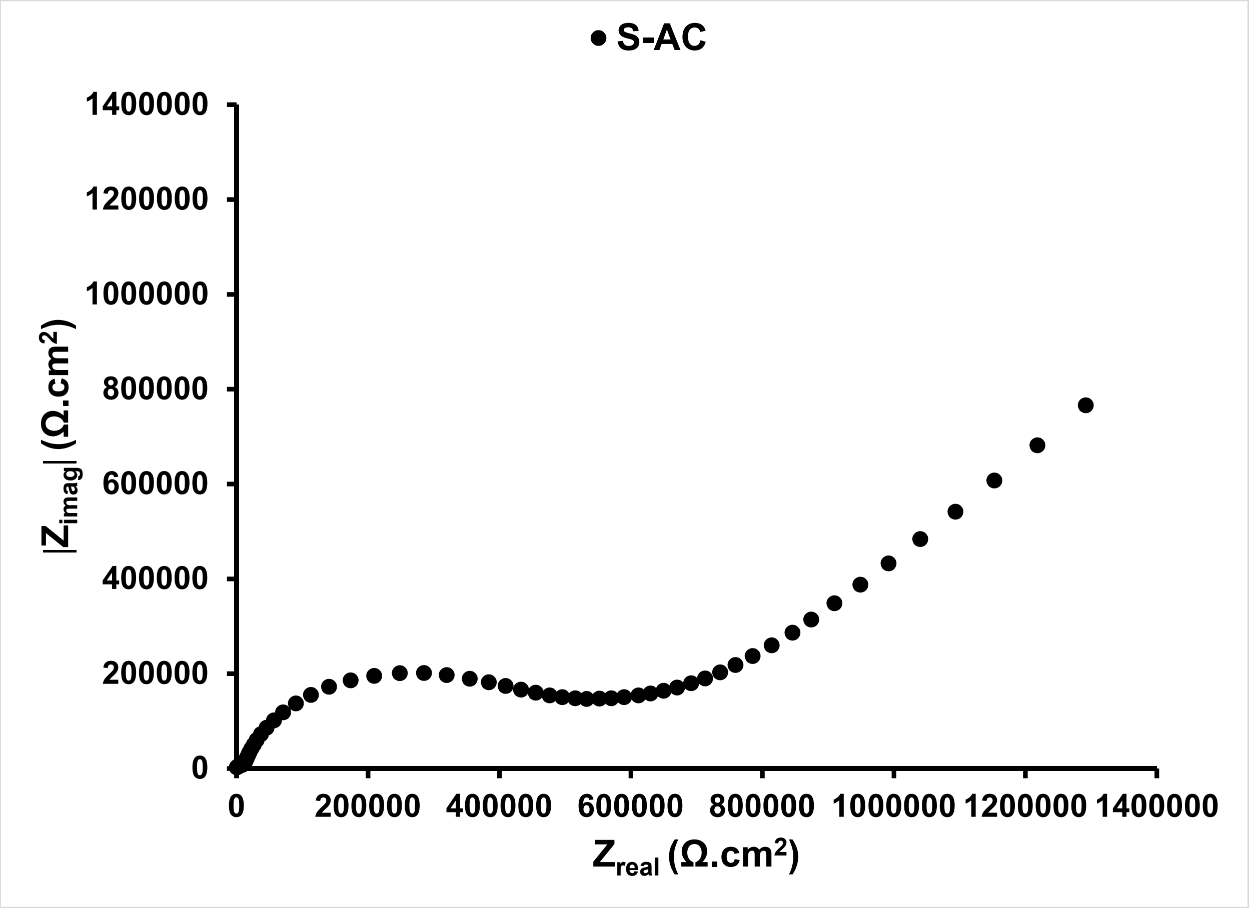

Figure S2. EIS Nyquist spectrum of **S-AC**-coated samples on AA3003 substrate after 30 days of exposure to the 3.5 wt.% NaCl solution.

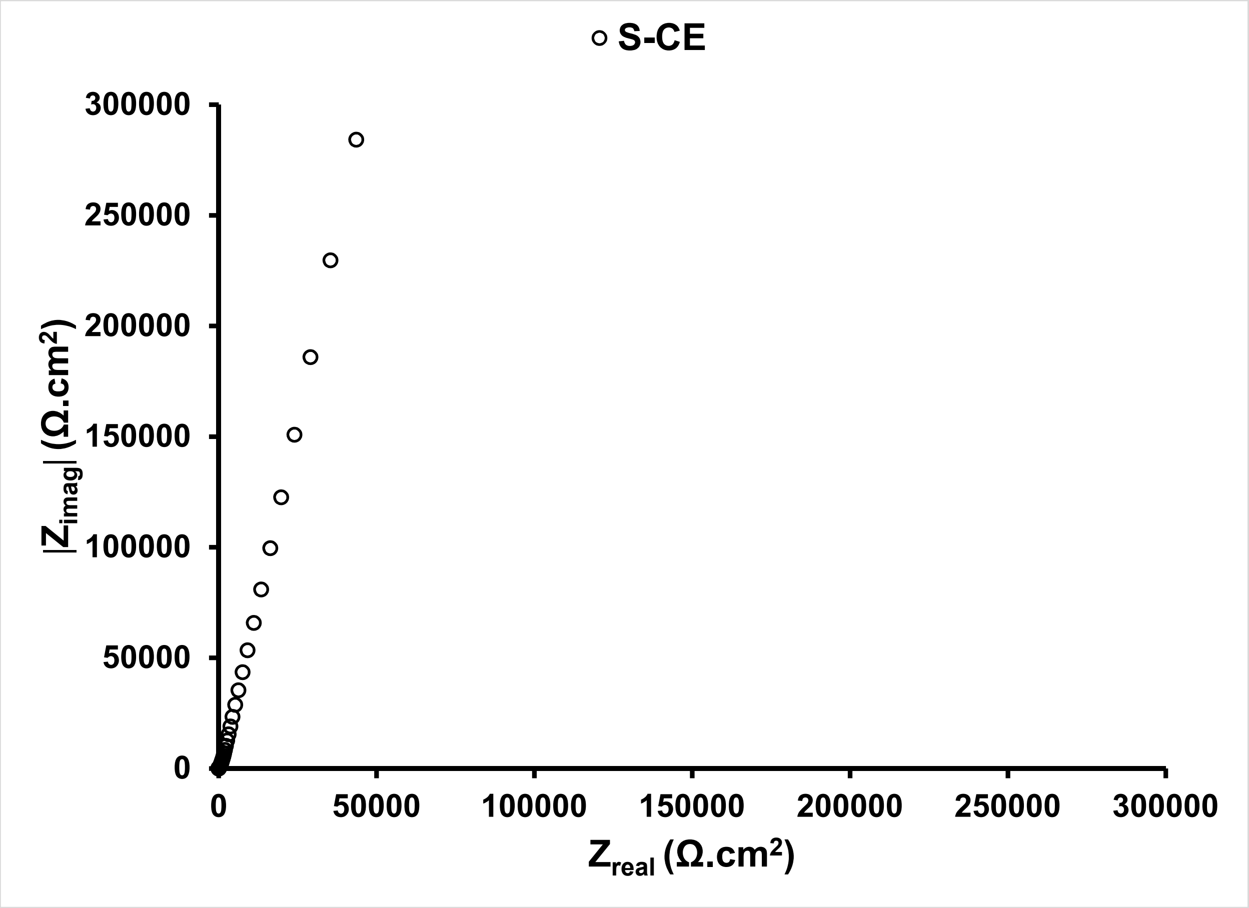

Figure S3. EIS Nyquist spectrum of **S-CE**-coated samples on AA3003 substrate after 30 days of exposure to the 3.5 wt.% NaCl solution.

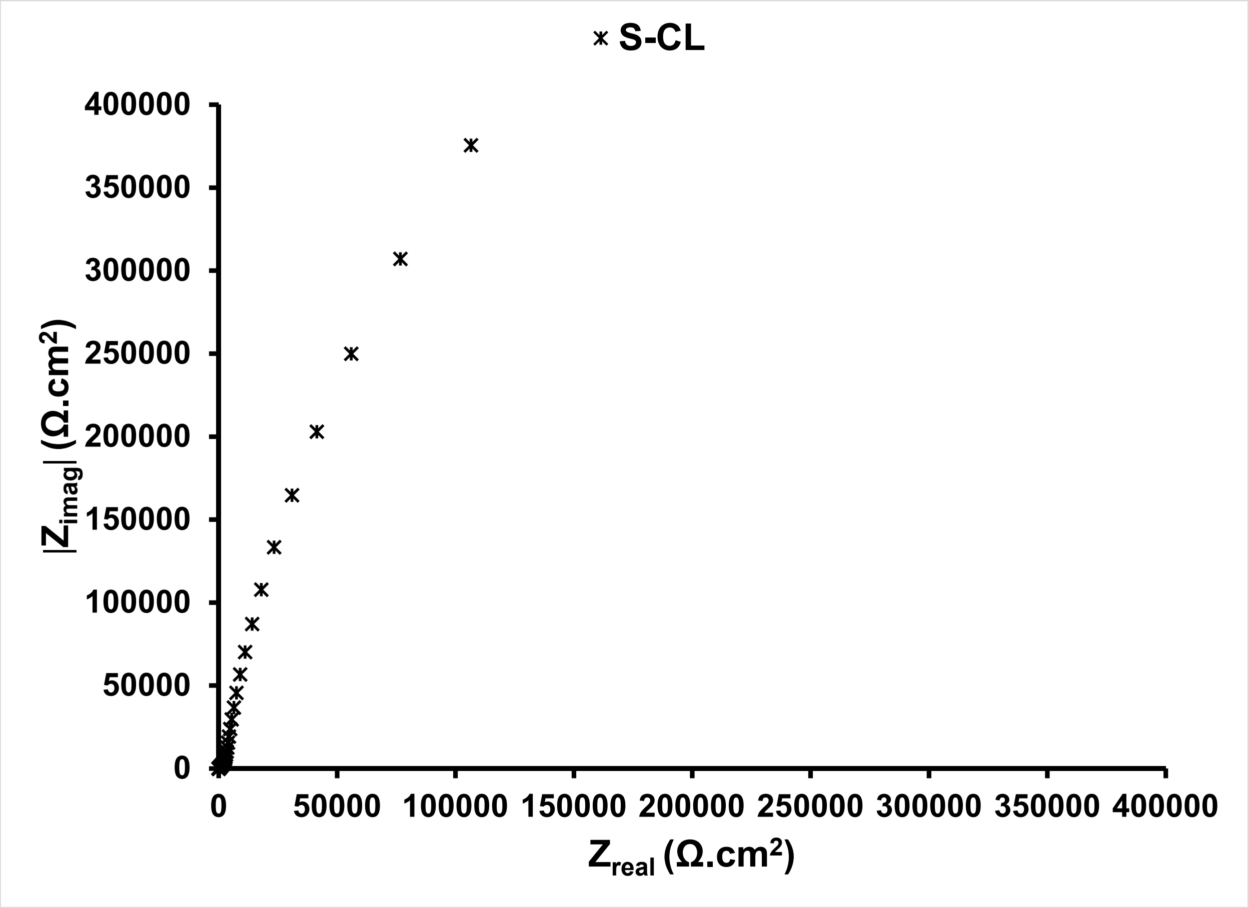

Figure S4. EIS Nyquist spectrum of **S-CL**-coated samples on AA3003 substrate after 30 days of exposure to the 3.5 wt.% NaCl solution.

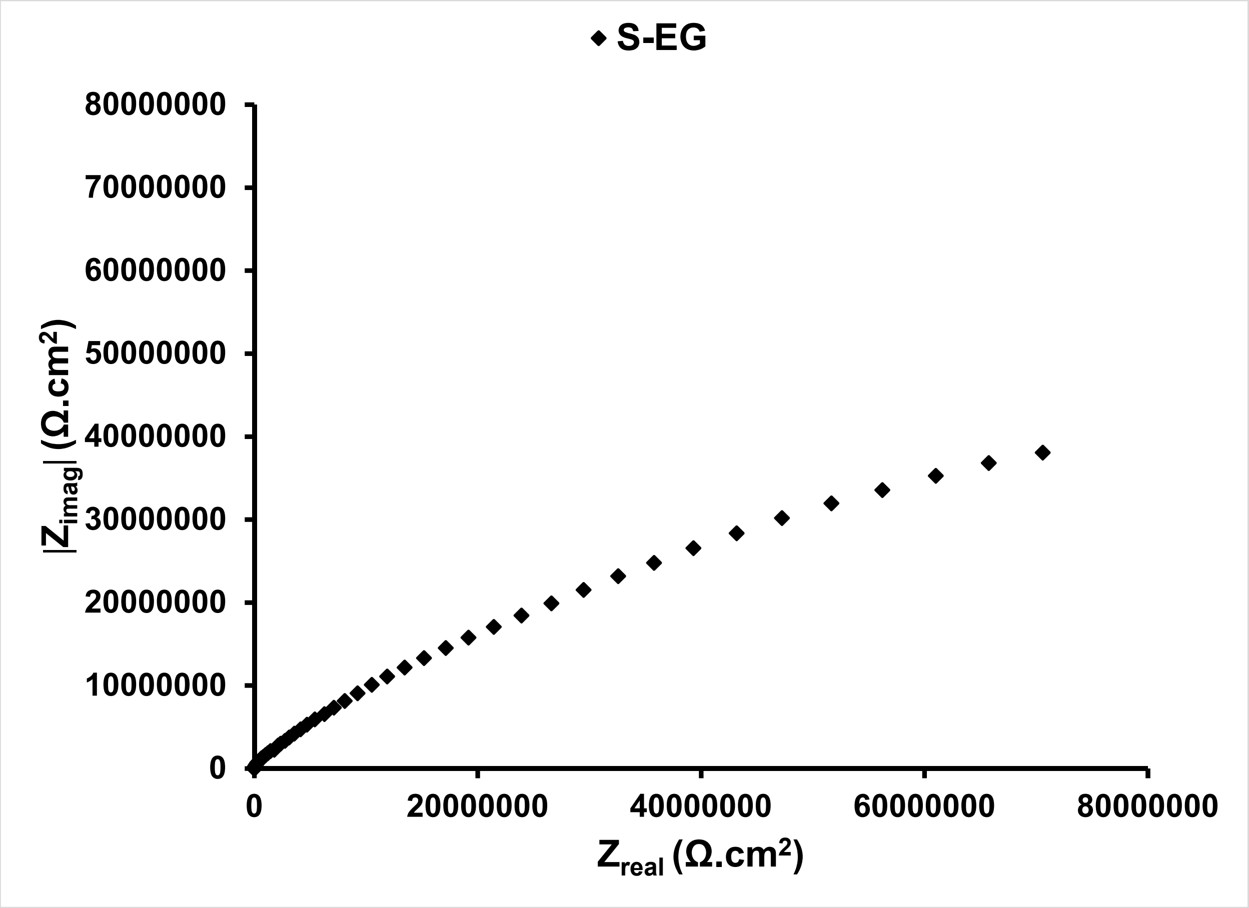

Figure S5. EIS Nyquist spectrum of **S-EG**-coated samples on AA3003 substrate after 30 days of exposure to the 3.5 wt.% NaCl solution.

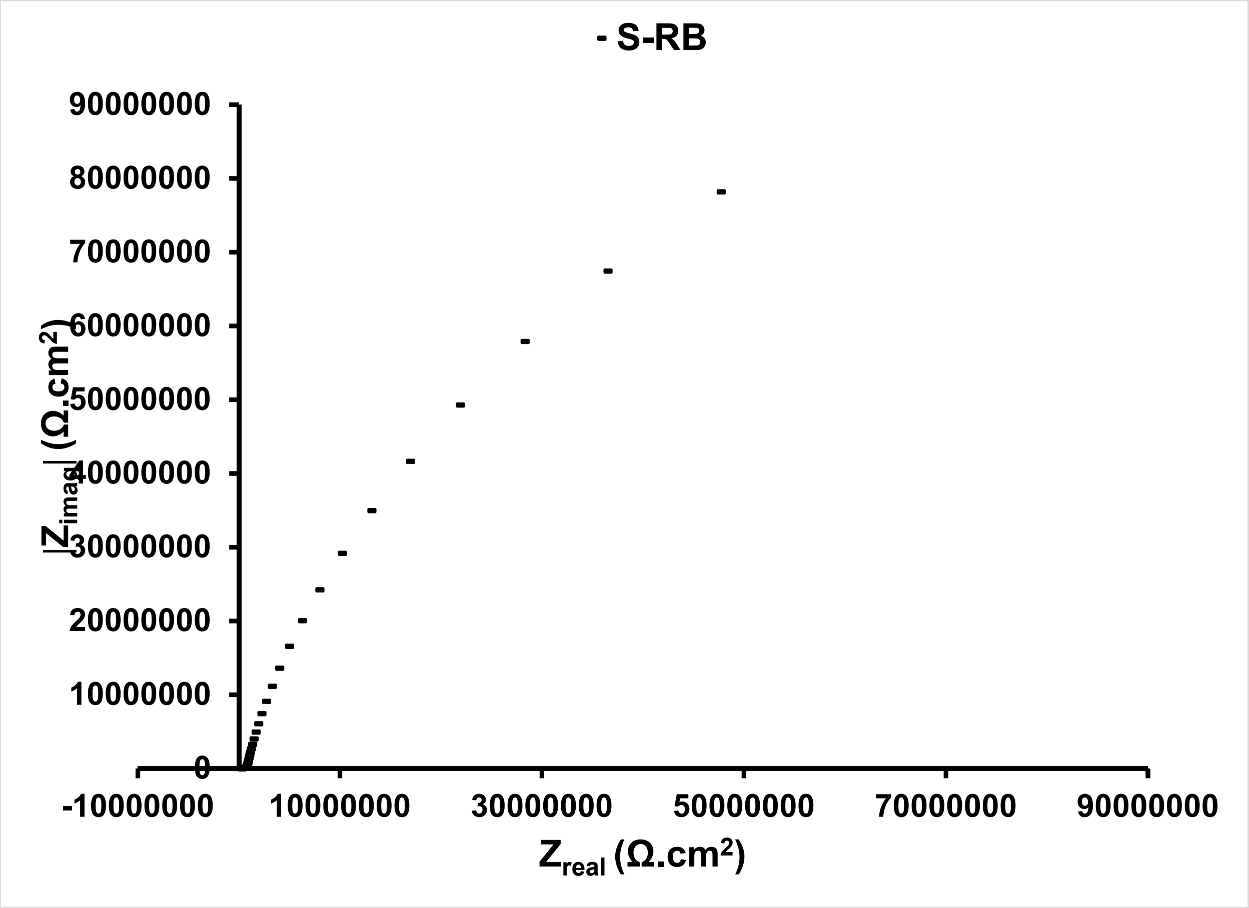

Figure S6. EIS Nyquist spectrum of **S-RB**-coated samples on AA3003 substrate after 30 days of exposure to the 3.5 wt.% NaCl solution.

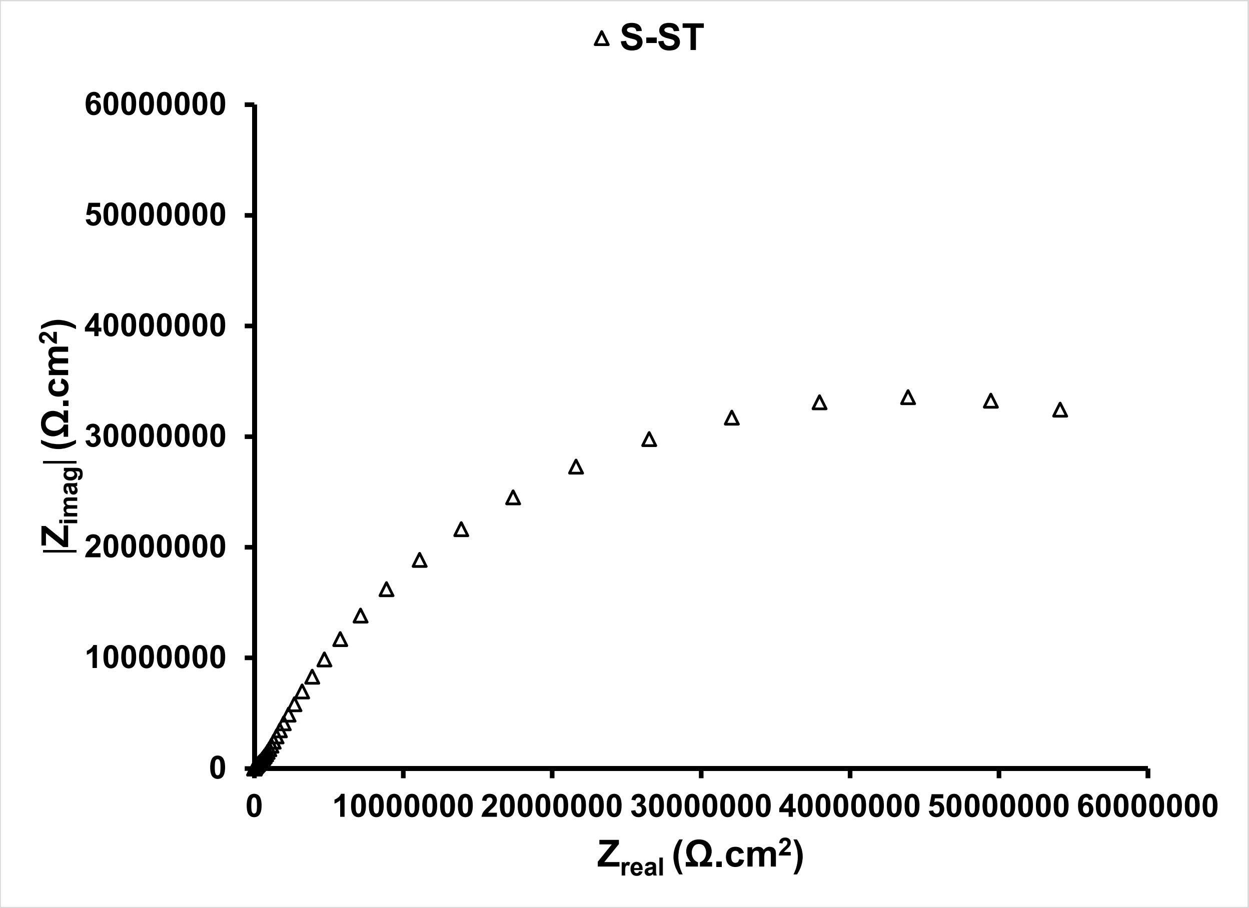

Figure S7. EIS Nyquist spectrum of **S-ST**-coated samples on AA3003 substrate after 30 days of exposure to the 3.5 wt.% NaCl solution.
